# The Gastric Juice

**Published:** 1847-09

**Authors:** 


*The Gastric Juice.*—Lehmann has confirmed the statements of Pelouze and of Bernard and Barreswill, regarding the absence of free hydrochloric acid, in the gastric juice, and has further indisputably proved the existence of free lactic acid in that secretion. To prevent any fallacy in the experiments, dogs were kept fasting for 12 or 16 hours, and then fed about a quarter of an hour before death with bones freed as much as possible from skin and fat. The gastric juice was almost perfectly clear, being scarcely opalescent. From 100 parts there were obtained 1·808 of solid matter, 0·25 of hydrochloric acid, and 98·097 of water. This hydrochloric acid is formed by the decomposing action of the lactic acid at a certain degree of concentration, even in the cold, upon certain chlorides, especially those of calcium and magnesium, but not those of potassium and sodium. To prove the presence of lactic acid itself with certainty, the gastric juice was concentrated *in vacuo* to one twelfth of its volume, the residue mixed with alcohol of 0·85, the spirituous solutions from several stomachs evaporated to the consistence of a syrup, and the residue exhausted with absolute alcohol. The residue of this was exhausted with ether, and the ethereal extract mixed with water, to remove the fat, and filtered. On further concentration, more drops of oil separated from the filtrate, and the fluid still contained hydrochlorate of ammonia.

The liquid was partly saturated with lime, partly with magnesia, and the salts formed were purified by several recrystallizations from alcohol and water. The magnesian salt gave results approximating very closely to the formula Mg O, La+3 HO."—*Ibid, from Dr. Day's Report on Chemistry.*

